# Ionizing Radiation Upregulates Glutamine Metabolism and Induces Cell Death via Accumulation of Reactive Oxygen Species

**DOI:** 10.1155/2021/5826932

**Published:** 2021-12-30

**Authors:** Pengfei Yang, Xiangxia Luo, Jin Li, Tianyi Zhang, Xiaoling Gao, Junrui Hua, Yonghong Li, Nan Ding, Jinpeng He, Yanan Zhang, Wenjun Wei, Jufang Wang, Heng Zhou

**Affiliations:** ^1^Key Laboratory of Space Radiobiology of Gansu Province & Key Laboratory of Heavy Ion Radiation Biology and Medicine, Institute of Modern Physics, Chinese Academy of Sciences, Lanzhou 730000, China; ^2^University of Chinese Academy of Sciences, Beijing 100049, China; ^3^Gansu Provincial Hospital of TCM, Lanzhou 730050, China; ^4^School of Nuclear Science and Technology, Lanzhou University, Lanzhou 730000, China; ^5^NHC Key Laboratory of Diagnosis and Therapy of Gastrointestinal Tumor, Gansu Provincial Hospital, Lanzhou 730000, China

## Abstract

Glutamine metabolism provides energy to tumor cells and also produces reactive oxygen species (ROS). Excessive accumulation of ROS can damage mitochondria and eventually lead to cell death. xCT (SLC7A11) is responsible for the synthesis of glutathione in order to neutralize ROS. In addition, mitophagy can remove damaged mitochondria to keep the cell alive. Ionizing radiation kills tumor cells by causing the accumulation of ROS, which subsequently induces nuclear DNA damage. With this in mind, we explored the mechanism of intracellular ROS accumulation induced by ionizing radiation and hypothesized new methods to enhance the effect of radiotherapy. We used MCF-7 breast cancer cells and HCT116 colorectal cancer cells in our study. The above-mentioned cells were irradiated with different doses of X-rays or carbon ions. Clone formation assays were used to detect cell proliferation, enzyme-linked immunosorbent assay (ELISA) detected ATP, and glutathione (GSH) production, while the expression of proteins was detected by Western blot and quantitative real-time PCR analysis. The production of ROS was detected by flow cytometry, and immunofluorescence was used to track mitophagy-related processes. Finally, BALB/C tumor-bearing nude mice were irradiated with X-rays in order to further explore the protein expression found in tumors with the use of immunohistochemistry. Ionizing radiation increased the protein expressions of ASCT2, GLS, and GLUD in order to upregulate the glutamine metabolic flux in tumor cells. This caused an increase in ATP secretion. Meanwhile, ionizing radiation inhibited the expression of the xCT (SLC7A11) protein and reduced the generation of glutathione, leading to excessive accumulation of intracellular ROS. The mitophagy inhibitor, or knockdown Parkin gene, is able to enhance the ionizing radiation-induced ROS production and increase nucleus DNA damage. This combined treatment can significantly improve the killing effect of radiation on tumor cells. We concluded that ionizing radiation could upregulate the glutamine metabolic flux and enhance ROS accumulation in mitochondria. Ionizing radiation also decreased the SLC7A11 expression, resulting in reduced GSH generation. Therefore, inhibition of mitophagy can increase ionizing radiation-induced cell death.

## 1. Introduction

The Warburg effect states that tumor cells consume large amounts of glucose for energy via the glycolytic pathway [1]; however, recent studies have shown that tumor cells rely heavily on glutamine metabolism to maintain cell survival rather than on glucose metabolism [2]. Glutamine is involved in cellular activities such as the preparation of nucleotides, the construction of DNA and RNA, and NADPH synthesis [3, 4]. Glutamine metabolism is considered to be the central metabolic pathway in tumor cells; this pathway contributes to oncogenic transformation [5] and promotes tumor growth [6]. Glutamine is transported to cells mainly via the alanine, serine, and cysteine-preferring transporter 2 (ASCT2) avenues [7]. It is then broken down into glutamic acid by glutaminase (GLS), which is then catalyzed by either glutamate dehydrogenase (GLUD) or aminotransferase to generate *α*-ketoglutaric acid, the molecule that can participate in oxidative phosphorylation or the tricarboxylic acid (TCA) cycle [8]. This increased glutamine consumption can enhance the ROS accumulation and reduce the antioxidant activity of tumor cells [9]. The glutamine metabolite also plays an important role in the promotion of cellular antioxidant defense. Glutamic acid can be used for biosynthesis of glutathione (GSH) [10]. xCT (SLC7A11) is a critical subunit of the amino acid antiporter system xc(-), which is involved in the synthesis of GSH [11]. xCT can transfer intracellular glutamic acid out of a cell and cysteine into the cell. This is a crucial mechanism by which tumor cells inhibit oxidative stress [12]. Inhibition of xCT resulted in reduced glutathione synthesis and an inability to respond to excessive accumulation of cellular peroxides, which ultimately led to cell death [13, 14]. In addition, studies have shown that tumor cells cannot survive if they run out of glutamine, a phenomenon called “glutamine addiction” [15, 16]. Under glutamine deprivation conditions, if additional metabolites related to TCA flux, such as succinic acid, fumaric acid, or malic acid, are added, they induce significant accumulation of lipid ROS in tumor cells [17, 18]. This indicated that although excessive glutamine intake can generate a large amount of energy through the TCA cycle, it also produces excessive ROS, which accelerates the process of intracellular lipid peroxidation and ultimately leads to cell death.

Mitochondria act as the energy factories of the cell. They produce energy to support cell survival. Oxidative phosphorylation and the TCA cycle are two of the processes that take place inside the mitochondria to provide energy. In the mitochondria, the oxidative phosphorylation process is accompanied by ROS production, so these organelles are especially vulnerable to oxidative damage. In this way, timely removal of damaged mitochondria is essential to cell survival [19]. Mitophagy is the targeted phagocytosis of damaged mitochondria via the lysosomal degradation pathway, which is the primary quality control mechanism for mitochondria [20, 21]. Depolarized mitochondria are enclosed by autophagosomes, and they combine with lysosomes to form autolysosomes. Eventually, the damaged mitochondria are degraded in the autolysosome [22]. Several studies have shown that Parkin plays a crucial role in mitophagy [23]. When mitochondria are damaged, PINK1 accumulates on the surfaces of the mitochondria and phosphorylates Parkin. In this way, Parkin is transported to the depolarized mitochondrial outer membrane, where it then recruits the P62 protein in order to promote the binding of the damaged mitochondria to the autophagosomes [24]. In tumor cells, mitophagy promotes both cell survival and therapeutic resistance [25]. In tumor stem cells, mitophagy degrades damaged mitochondria and reduces intracellular ROS accumulation, which help to maintain cell differentiation. Mitophagy also plays an antiapoptotic role in liver tumor cells [26, 27]. In this way, blocking mitophagy can reduce the therapeutic resistance of tumor cells and promote cell death.

Radiotherapy is a commonly used tumor treatment method that can induce a variety of cell death modes, such as apoptosis, necrosis, and ferroptosis. Ionizing radiation leads to DNA damage and causes the increase in intracellular ROS accumulation [28]. ROS then attack proteins, fatty acids, nucleotides, and other molecules, which initiates the peroxidation reaction and leads to cell death [29]. Ionizing radiation can also induce mitophagy, which helps maintain the dynamic balance of oxidative phosphorylation in the mitochondria. This is one reason that tumor cells become resistant to radiotherapy. Studies have shown that the mitophagy inhibitor temozolomide-perillyl alcohol conjugate (TMZ-POH) induces lysosome dysfunction in nonsmall cell lung cancer (NSCLC) cells and then destroys mitophagic function and improves tumor radiosensitivity, resulting in a reduced cell survival rate [30]. Based on this investigation, we hypothesized that ionizing radiation could induce excessive glutamine intake and thus cause ROS overproduction and increase lipid peroxidation levels to induce cell death. We also examined the effects of the inhibition of mitophagy and whether this process could promote ionizing radiation-induced cell death. The results of this study may provide a reference for further research into improving the effectiveness of radiotherapy.

## 2. Materials and Methods

### 2.1. Radiotherapy

The X-ray was generated by an X-Rad 225 generator (Precision, North Branford, CT, US) with an energy of 225 KV/13.3 mA. Carbon ion (^12^C^6+^) beam radiation was performed at the treatment terminal of the Institute of Modern Physics with an energy of 80 MeV/u, peak LET: 50 KeV/*μ*m, SOBP. The cells or breast tumor-bearing nude mice were placed on a platform after the mice were anesthetized (pentobarbital sodium (30 mg/kg), intraperitoneal). The bodies of the mice were protected with a lead plate, and only the tumor site was exposed to radiation.

### 2.2. The Cell Lines

Breast cancer cells (MCF-7) and colorectal cancer cells (HCT116) were purchased from the Chinese Academy of Sciences Cell Bank. Cells were cultured in Dulbecco's Modified Eagle Medium (DMEM) containing 10% fetal bovine serum (FBS) and 100 U/mL penicillin and 100 *μ*g/mL streptomycin. The culture was maintained at 37°C and 5% CO_2_ in a constant temperature incubator with saturated humidity.

### 2.3. Experiments on Mice Models

Six-week-old female BALB/C nude mice were purchased from Xi'an Keao Biological Technology Co. Ltd. and maintained in the animal facility at the Institute of Modern Physics under specific pathogen-free conditions in a temperature-controlled environment with 12 h of light and 12 h of dark cycles. Mice received food and water ad libitum. All of the animal experiments were approved by the Ethical Committee of the Institute of Modern Physics and followed the EU Directive 2010/63/EU guidelines. MCF-7 tumors were established in BALB/C nude mice hosts by subcutaneously inoculating 1,000,000 cells. When the tumors became palpable at about 50 mm^3^, they were locally irradiated with 2 Gy X-ray.

### 2.4. Western Blot

The cells were collected by RIPA lysate, containing a protease inhibitor and a phosphatase inhibitor, and then crushed with ultrasonic. Then, samples were mixed with 5× loading buffer. The protein was placed in a 98°C metal bath and heated for 10 min in order to denature the protein. For analysis, we used constant pressure electrophoresis. The voltage was set at 80 V for protein concentration and at 120 V for separation. The membrane was transferred at 120 V and 0.2 A, although the specific transfer time depended on the molecular weight. Samples were then incubated with 5% BSA at room temperature for 2 h. The primary antibody was incubated overnight at 4°C. The next day, after washing five times with PBST, an HRP-labeled secondary antibody was incubated at room temperature for 90 min. Images were obtained by electrochemiluminescence immunoassay and analyzed with Image J software [31]. All antibody information is presented in Supplementary Table 1.

### 2.5. Real-Time Quantitative PCR

We used the Trizol method to extract RNA, and cDNA was obtained by conducting reverse transcription of 3 *μ*g of RNA according to the GeneCopopoeia (Rockville, MD, US) reverse transcription kit manual (Cat: AORT-0020). The cDNA was diluted three times, and the SYBR Green system was used for real-time quantitative detection. The final volume was 20 *μ*L, with a total of 40 cycles. Settings were as follows: predenaturation at 95°C for 10 min, denaturation at 95°C for 10 s, annealing at 60°C for 20 s, and extension at 72°C for 15 s, and the heating rate from 72°C to 95°C was 0.5°C/6 s. All data were collected by the software of the Bio-Rad CFX96 PCR system. The collected Ct values were calibrated with GAPDH as an internal reference. The target gene expression was analyzed using the ^2−∆^CT method [31]. Primers and sequences are listed in Supplementary Table 2.

### 2.6. Flow Cytometry

The cells were digested with trypsin and collected in a medium containing 5% serum. Single-cell suspensions were prepared by gentle teasing through disposable 70 *μ*m sieves into cold PBS. Then, cells were spun at 800 rpm for 10 min. The supernatant was discarded, and 1 × 10^6^ cells were resuspended in the cell staining buffer (#420201, Biolegend) containing dihydrorhodamine 123 (DHR123). Cells were then incubated in the dark at room temperature for 10 min. ROS expression was detected using a Merck Millipore FlowSight flow cytometer.

### 2.7. ELISA Assay

MCF-7/HCT-116 cells were cultured in a dish, and the supernatant medium was collected after irradiation for 48 h. Following the instructions provided in the Luminescent ATP Detection Assay Kit (Abcam, AB113849), ATP was measured at a spectrophotometer wavelength of 450 nm. Following the instructions provided in the Micro Reduced Glutathione Assay Kit (Solarbio, BC1175), GSH was measured at a spectrophotometer wavelength of 412 nm.

### 2.8. Immunofluorescence

For live image collection, cells were incubated in the medium mixed with Mito-Tracker Green (100 nM) and Lyso-Tracker Red (60 nM) in the dark for a total of 10 min. Then, Hoechst 33342 (1 *μ*m) was added, and cells were further incubated at room temperature for 10 min. Images were obtained using a fluorescence microscope.

For fixed image collection, 4% paraformaldehyde was used to fix cells for about 10 min. Then, the supernatant was discarded, and cells were incubated with cold methanol for 20 min. Finally, the cells were prepared by cleaning with 75% glacial ethanol. After rehydration, 0.5% Triton X-100 was used to permeate the cell membrane for about 5 min. The samples were then placed in goat serum diluted with PBS (1 : 20) and blocked for 2 h. This was followed by the addition of primary antibody at a dilution ratio of 1 : 500 and incubation for an additional 2 h. After rinsing with PBST five times, the corresponding fluorescent secondary antibody at a dilution ratio of 1 : 1000 was incubated in the dark for 1.5 h. Then, 10 *μ*l of DAPI was added, and the images were captured by a fluorescence microscope [31].

### 2.9. Immunohistochemical

The tumor was embedded in paraffin and cut into 3 *μ*m slices. After dewaxing and hydration, the slices were put in 0.01-M sodium citrate buffer solution at pH 6.0 and heated at 95°C for 10 min. Then, 0.5% Triton X-100 was used to permeate the cell membrane, and 3% H2O2 was added to remove endogenous peroxides. After blocking, the primary antibody against Ki67 (1 : 500) and MDA (1 : 500) was incubated overnight with conditions maintained at 4°C. On the second day, after PBST washing, biotin was labeled with secondary antibodies. The antibody was incubated at room temperature for 30 min. Then, DAB staining and hematoxylin redyeing were performed.

### 2.10. Data Statistics and Analysis

Origin 9.0 was used to analyze the results, and the data were represented as *x* ± SE. Student's *t*-test, one-way ANOVA with post hoc Dunnett-test, one-way repeated ANOVA test, and Kaplan-Meier analysis with Log-Rank-test tested significant differences, and ^∗^*p* < 0.05, ^∗∗^*p* < 0.01, and ^∗∗∗^*p* < 0.001 were considered to be statistically significant. Each experiment was performed at least three times independently.

## 3. Results and Discussion

### 3.1. Results

#### 3.1.1. Ionizing Radiation Inhibits Cell Proliferation

**Figure 1:**
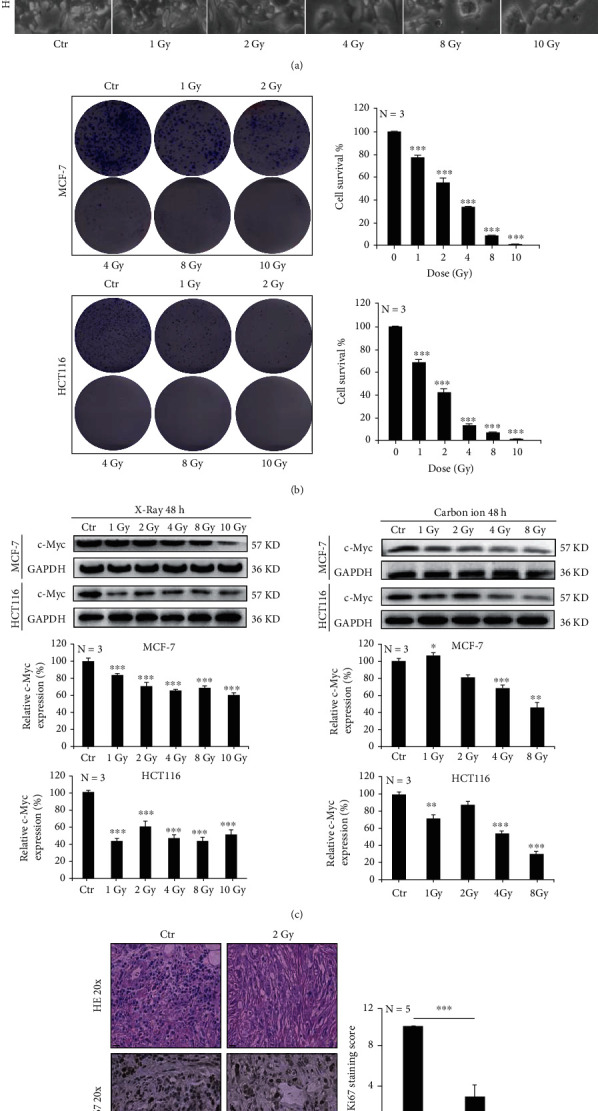
Ionizing radiation inhibits cell proliferation. MCF-7 and HCT-116 cells were irradiated with X-rays at different doses. At 48 h after irradiation, the morphology of cells became swollen (a). Clonal formation was inhibited by radiation in a dose-dependent manner (b). The expression of c-Myc protein was decreased after irradiation with different doses of X-rays and carbon ions (c). 2 Gy X-ray irradiation reduced the malignancy index of the MCF-7 tumor cells (d). Mean ± SD of triplicate assessments, one-way ANOVA with post hoc Dunnett test, ^∗^*p* < 0.05, ^∗∗^*p* < 0.01, and ^∗∗∗^*p* < 0.001 (b, c). Student's unpaired *t*-test, ^∗∗∗^*p* < 0.001 (d).

Breast cancer cells (MCF-7) and colorectal cancer cells (HCT116) were irradiated with different doses of X-rays. We found that the cellular morphology became swollen and flat at 48 h after irradiation, and the number of cells decreased (Figure 1(a)). The clone formation was measured at 15 days after irradiation. The number of clones reduced after irradiation in a dose-dependent manner, and the dots formed by clones appeared smaller (Figure 1(b)). In addition, we also irradiated tumor cells with X-rays and a carbon ion beam. Results showed that ionizing radiation significantly reduced the expression of the malignant proliferative protein C-myc (Figure 1(c)). In the *in vivo* experiment, 2 Gy X-rays were used to irradiate the tumor site of MCF-7 tumor-bearing nude mice. After 10 days of irradiation, the tumor was harvested and paraffin-embedded. We found that many cells in the tumor tissue died after hematoxylin-eosin staining, and Ki67 staining showed that the malignant proliferation of tumor cells was significantly decreased (Figure 1(d)). These results indicate that ionizing radiation can dramatically inhibit cell's malignant proliferation.

#### 3.1.2. Ionizing Radiation Upregulates Glutamine Metabolism in Tumour Cells

**Figure 2:**
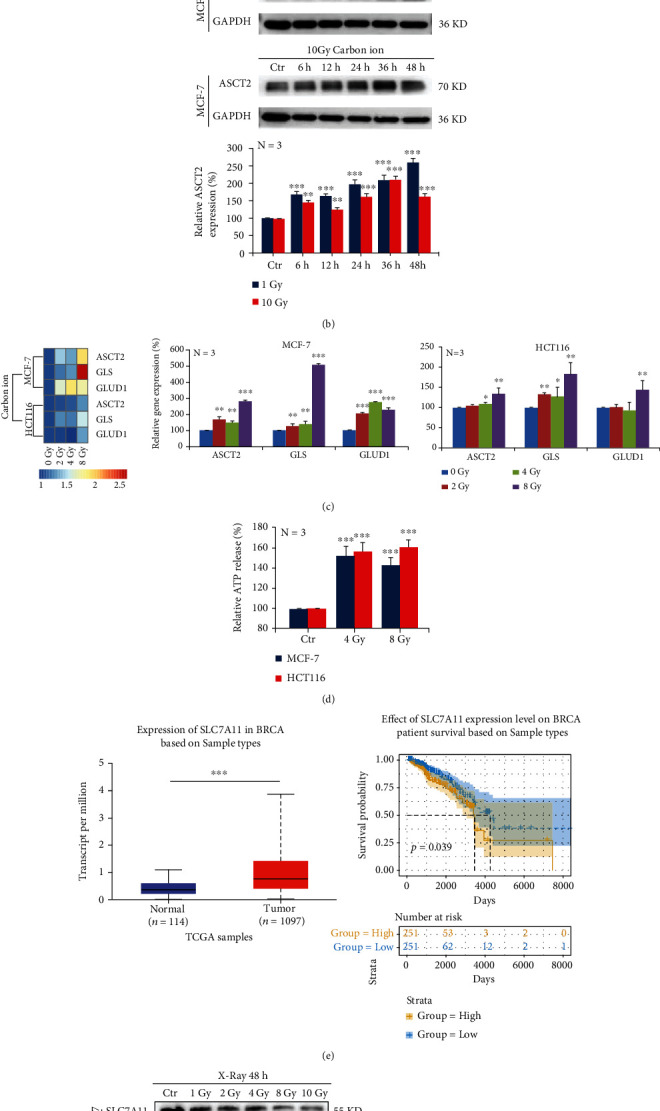
Ionizing radiation upregulates glutamine metabolism in tumor cells. MCF-7 cells were irradiated with 1 Gy and 10 Gy carbon ions. After 2 days, we performed an untargeted metabolome analysis and found that (a) the protein expression of ASCT2 increased in a time-dependent manner under carbon ion irradiation (b). Carbon ions could upregulate the mRNA expressions of ASCT2, GLS, and GLUD in MCF-7 cells, by real-time quantitative PCR detection (c). X-rays induced the increase of extracellular ATP secretion in MCF-7 cells, by ELISA detection (d). SLC7A11 gene was analyzed based on sample types in the TCGA database (e). The protein expression of SLC7A11 decreased in a dose-dependent manner under X-ray irradiation (f). ELISA detection revealed that X-ray irradiation reduced the GSH production in MCF-7 cells (g). Mean ± SD of triplicate assessments, one-way repeated ANOVA test, ^∗∗^*p* < 0.01, and ^∗∗∗^*p* < 0.001 (b). One-way ANOVA with post hoc Dunnett test, ^∗^*p* < 0.05, ^∗∗^*p* < 0.01, and ^∗∗∗^*p* < 0.001 (c, d). Kaplan-Meier analysis with log-rank test, ^∗^*p* < 0.05, ^∗∗^*p* < 0.01, and ^∗∗∗^*p* < 0.001 (e). One-way ANOVA with post hoc Dunnett test, ^∗^*p* < 0.05, ^∗∗^*p* < 0.01, and ^∗∗∗^*p* < 0.001 (f, g).

In order to explore the reasons for the inhibition of cell proliferation, we explored whether ionizing radiation inhibited the energy supply of tumor cells from the perspective of glutamine metabolism. We irradiated MCF-7 cells with 1 Gy and 10 Gy carbon ion beams and found that the glucose metabolic flux was decreased, while the trichloroacetic acid (TCA) flux was increased considerably (Figure 2(a)). Meanwhile, Western blot analysis also confirmed that the carbon ion beam could upregulate the protein expression of ASCT2 (Figure 2(b)). In addition, we irradiated MCF-7 and HCT116 cells with 2 Gy, 4 Gy, and 8 Gy carbon ion beams and found that the mRNA expression of ASCT2, GLS, and GLUD increased 48 h after exposure (Figure 2(c)). We also found that ATP production increased at 48 h after 4 Gy and 8 Gy X-ray irradiation in MCF-7 and HCT116 cells by ELISA kit detection (Figure 2(d)).

Furthermore, we analyzed the TCGA database and found that cancer patients with high SLC7A11 gene expression had a lower survival rate, which supports the idea that high SLC7A11 expression is usually an indicator of poor patient prognosis (Figure 2(e)). The protein expression of SLC7A11 decreased at 48 h after irradiation with different doses of X-rays in both MCF-7 and HCT116 cells (Figure 2(f)). In addition, we detected a significant decrease in GSH secretion in MCF-7 and HCT116 cells at 48 h after X-ray irradiation (Figure 2(g)). These results indicated that ionizing radiation did not inhibit the energy supply of tumor cells but upregulated the metabolic flux of glutamine instead.

#### 3.1.3. Ionizing Radiation Enhances Oxidative Stress in Tumor Cells

**Figure 3:**
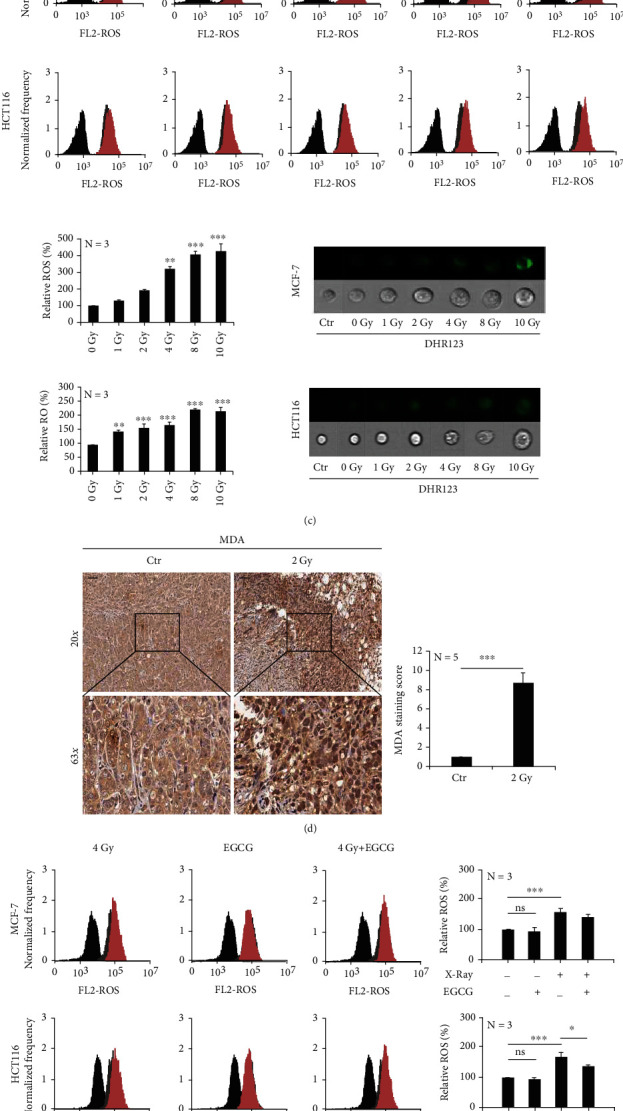
Ionizing radiation enhances oxidative stress in tumor cells. MCF-7 and HCT-116 cells were irradiated with X-rays at different doses. The morphology of the mitochondria of MCF-7 cells was observed by transmission electron microscopy (a). Immunofluorescence staining showed that X-rays caused mitochondrial shrinkage (b). DHR123 staining showed that X-rays induced ROS accumulation in mitochondria in a dose-dependent manner (c). 2 Gy X-ray irradiation increased the peroxidation index of the MCF-7 tumor cells (d). Inhibition of glutamine metabolic flux reduces the accumulation of ROS (e) and reduces DNA damage (f, g) induced by ionizing radiation. Mean ± SD of triplicate assessments, one-way ANOVA with post hoc Dunnett test, ^∗∗^*p* < 0.01, and ^∗∗∗^*p* < 0.001 (b, c). Student's unpaired *t*-test, ^∗∗∗^*p* < 0.001 (d). One-way ANOVA with post hoc Dunnett test, ^∗^*p* < 0.05, ^∗∗^*p* < 0.01, and ^∗∗∗^*p* < 0.001 (e–g).

According to the above results, ionizing radiation could induce tumor cells to produce more ATP, but cell proliferation was still inhibited. Therefore, we focused on the energy factory mitochondria to explore the mechanism. We irradiated MCF-7 and HCT116 cells with 4 Gy and 8 Gy X-rays. After 48 h, the mitochondria swelled, became deformed, and cristae broke in MCF-7 cells (Figure 3(a)). Subsequently, we used Tomm20 antibody to label mitochondria, and immunofluorescence results showed that both branched and unbranched cristae were smaller in MCF-7 and HCT116 cells after X-ray irradiation (Figure 3(b)).

In addition, some cells were labeled dihydrorhodamine 123 (DHR123). We found via flow cytometry that ROS has accumulated in an irradiation dose-dependent manner in the mitochondria (Figure 3(c)). In animal experiments, 2 Gy X-rays irradiated the MCF-7 tumor site, and we found by immunohistochemical analysis that the lipid peroxidation (MDA) was significantly increased (Figure 3(d)). In order to verify the relationship between the increased glutamine metabolic flux and the accumulation of ROS in mitochondria, which was induced by ionizing radiation, we irradiated MCF-7 and HCT116 cells, in combination with the glutaminase inhibitor epigallocatechin gallate (EGCG). We found that the combination treatment significantly decreased ROS accumulation (Figure 3(e)), and *γ*H2Ax staining and Western blot detection revealed that the nuclear DNA damage was also alleviated (Figures 3(f) and 3(g)). These results indicated that ionizing radiation induced intracellular ROS overaccumulation and enhanced cellular oxidative stress.

#### 3.1.4. Ionizing Radiation Induces Mitophagy in Tumor Cells

**Figure 4:**
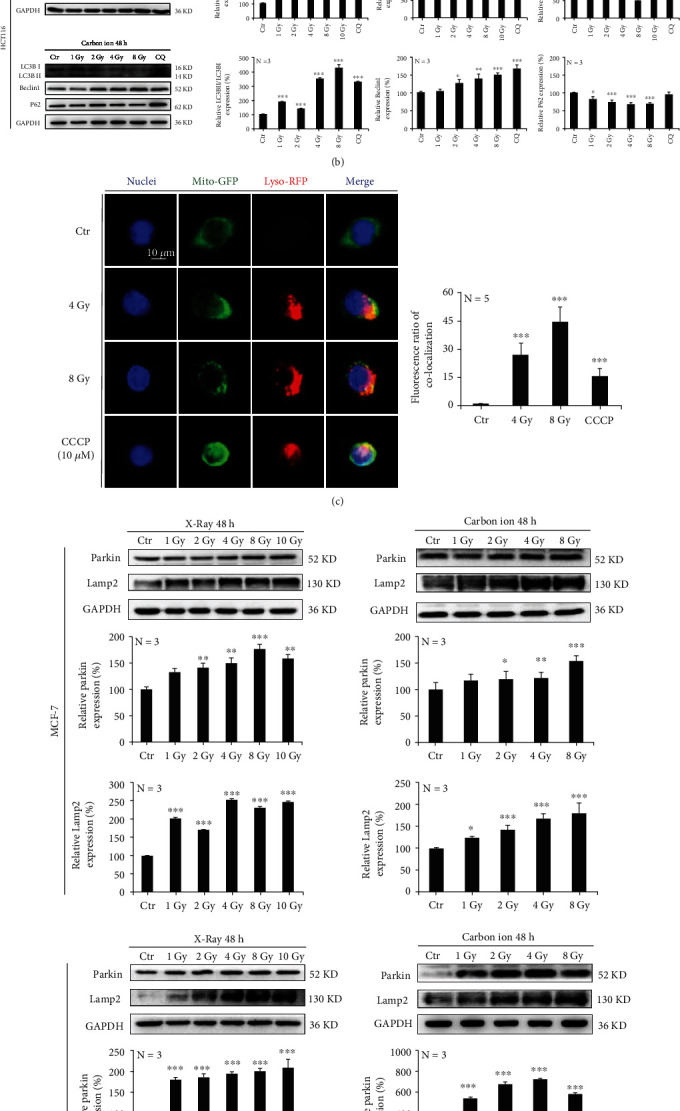
Ionizing radiation induces mitophagy in tumor cells. MCF-7 and HCT-116 cells were irradiated with X-rays at different doses. The acidity of cells was detected by flow cytometry with acridine orange staining (a). The expression of autophagy-associated proteins LC3B and Beclin 1 was increased, while that of p62 was decreased (b). X-rays increased the mitochondrial and lysosome colocalization (c). The expression of mitophagy-associated proteins Parkin and LAMP2 was increased in a dose-dependent manner (d). MCF-7 cells were irradiated with X-rays at different doses, and this induced the increased expression of cytochrome C (e) and very mild apoptosis at 24 h after irradiation (f). Mean ± SD of triplicate assessments, one-way ANOVA with post hoc Dunnett test, ^∗^*p* < 0.05, ^∗∗^*p* < 0.01, and ^∗∗∗^*p* < 0.001 (a–f).

Ionizing radiation caused excess ATP production in tumor cells, which resulted in ROS-induced oxidative stress in the mitochondria. Knowing this, we wanted to explore how mitochondria respond to oxidative stress. MCF-7 and HCT116 cells were irradiated with 1 Gy, 2 Gy, 4 Gy, 8 Gy, and 10 Gy of X-rays. After 48 h, acridine orange staining via flow cytometry detection revealed that the acidity of cells was significantly increased (Figure 4(a)). Results indicated that the expression of LC3B and Beclin 1 proteins was increased, while the expression of p62 was decreased (Figure 4(b)).

Furthermore, MCF-7 cells were irradiated with 4 Gy and 8 Gy X-rays. After 48 h, mitochondria and lysosomes were labeled using Mito-Tracker Green and Lyso-Tracker Red, respectively. We found that ionizing radiation induced a significant increase in mitochondrial and lysosome colocalization (Figure 4(c)). Subsequently, the expression of Parkin and LAMP2, a lysosomal marker protein, was also significantly increased in an irradiation dose-dependent manner (Figure 4(d)). Although X-rays were found to increase the protein expression of cytochrome c in MCF-7 cells (Figure 4(e)), only mild apoptosis was detected by flow cytometry (Figure 4(f)). According to the above results, mitochondria activated mitophagy in order to resist the oxidative stress caused by ionizing radiation.

#### 3.1.5. Inhibition of Mitophagy Promotes Ionizing Radiation-Induced Cell Death

Radiotherapy typically induces ROS accumulation in tumor cells, which causes DNA strand damage and leads to cell death. Therefore, we wanted to investigate whether cell death can be improved by preventing ROS clearing with the use of a mitophagy inhibitor.

**Figure 5:**
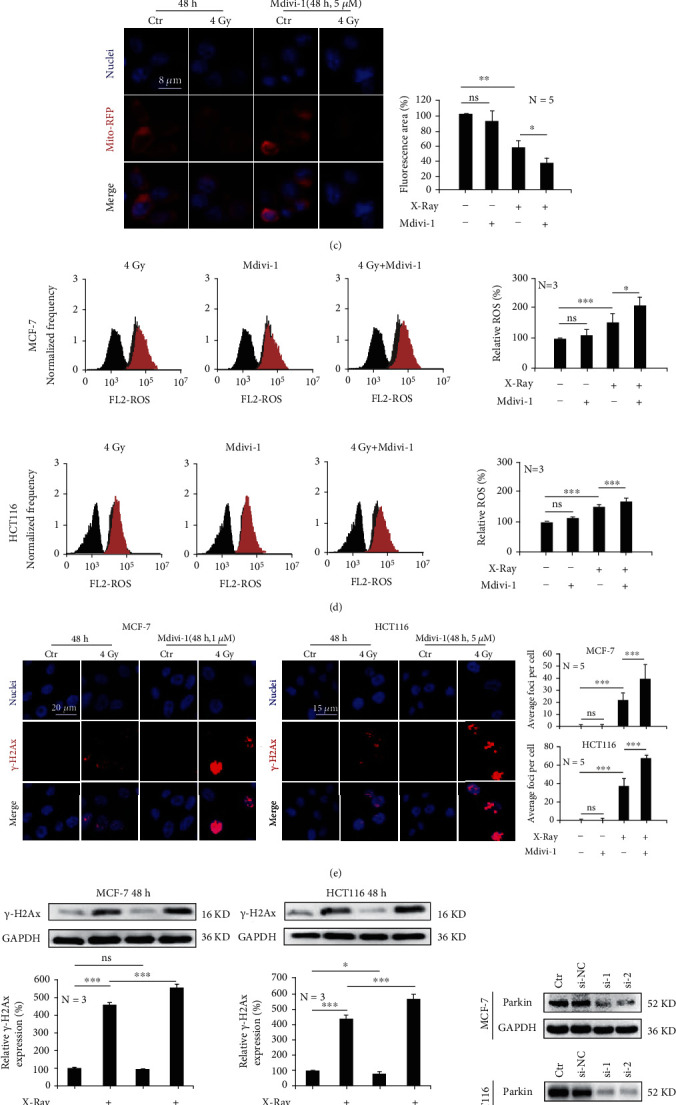
Inhibition of mitophagy promotes ionizing radiation-induced cell death. MCF-7 and HCT-116 cells were irradiated with 4 Gy and/or 8 Gy X-rays. Mdivi-1 decreased X-ray-induced expression of mitophagy-related proteins (a, b) and the mitochondrial membrane potential (c). Mdivi-1 led to increased ROS accumulation (d) and increased DNA damage (e, f). Silencing the Parkin gene (g) led to increased ROS accumulation induced by X-rays (h). Mdivi-1 or silencing the Parkin gene enhanced cell death induced by X-rays (i, j) and reduced clone formation (k). Mean ± SD of triplicate assessments, one-way ANOVA with post hoc Dunnett test, ^∗^*p* < 0.05, ^∗∗^*p* < 0.01, and ^∗∗∗^*p* < 0.001 (a–k).

MCF-7 and HCT116 cells were irradiated with X-rays and combined with Mdivi-1 treatment, which functioned as the mitophagy inhibitor. We found that branched and unbranched cristae in the mitochondria became smaller; however, immunofluorescence detection revealed that Parkin expression was significantly reduced in MCF-7 cells (Figure 5(a)). We also found that Mdivi-1 significantly downregulated the protein expressions of Parkin, LAMP2, and LC3 induced by ionizing radiation in MCF-7 and HCT116 cells (Figure 5(b)). In addition, Mdivi-1 further downregulated mitochondrial membrane potential (Figure 5(c)) and caused increased ROS accumulation after ionizing radiation in both MCF-7 and HCT-116 cells (Figure 5(d)). Ultimately, the combination treatment led to more nuclear DNA damage (Figures 5(e) and 5(f)). When the Parkin gene was knocked down in MCF-7 and HCT116 cells (Figure 5(g)), X-rays were able to significantly induce ROS production (Figure 5(h)). The use of Mdivi-1 and the knockdown of the Parkin gene significantly increased X-ray-induced cell death (Figures 5(i) and 5(j)) and reduced clone formation (Figure 5(k)). Based on these results, it can be concluded that the inhibition of mitophagy can increase the accumulation of ROS and induce an increased level of cell death.

## 4. Discussion

Ionizing radiation downregulated the glucose metabolic flux of cells, which in turn upregulated the glutamine metabolic flux in order to provide sufficient energy to maintain survival. Our study showed that high glutamine metabolic flux induces the production of a large amount of *α*-ketoglutaric acid, which greatly increases the productivity of the TCA cycle and leads to the accumulation of a large amount of ROS. The expression of SLC7A11 in MCF-7 and HCT116 cells decreased after ionizing radiation, leading to reduced glutathione synthesis and the inability to consume the ROS that had been produced by the TCA cycle. This resulted in oxidative stress injury to cells and ultimately led to cell death.

It has been previously reported that a high glutamine metabolic flux can inhibit the apoptosis of tumor cells [32]. In our study, it was also confirmed that the rate of apoptosis in MCF-7 cells was only about 10% after 4 Gy X-ray irradiation; however, the expression of cytochrome c was elevated. We believe that the release of cytochrome c may be associated with decreased mitochondrial membrane potential because this also induced mitophagy and then cleared the damaged mitochondria. Cytochrome c may help maintain cell survival instead of apoptosis. Studies have shown that the upregulation of the c-Myc gene can increase the metabolic flux of glutamine in tumor cells [33]. In our study, ionizing radiation downregulated the expression of c-Myc and upregulated the metabolic flux of glutamine. This indicates that the high flux of glutamine metabolism induced by ionizing radiation does not depend on the regulation of the c-Myc gene.

The wide application of stereotactic body radiotherapy improves the accuracy and safety of clinical radiotherapy and dramatically reduces the damage to normal tissues [34]. Although high doses of ionizing radiation induce cell death, cells can also change their fate through other mechanisms, such as mitophagy [35]. ROS production induced by ionizing radiation can cause autophagy as a stress response, and excessive accumulation of ROS can cause mitophagy. Our study confirmed that ionizing radiation could significantly induce increases in Parkin expression. Mitophagy not only saves a cell by clearing out damaged mitochondria but also reduces the effectiveness of radiotherapy for cancer patients. Blocking mitophagy may help inhibit the self-rescue mechanism of tumor cells. We further irradiated MCF-7 and HCT116 cells and combined these cells with the mitophagy inhibitor Mdivi-1. Results showed that cell death increased significantly. We further demonstrated that inhibition of mitophagy by knockdown of the Parkin gene could markedly reduce ionizing radiation-induced cell death. We therefore believe that targeting Parkin can help regulate cell death, so it could be a new radio sensitization target in tumor radiotherapy.

## 5. Conclusions

In conclusion, ionizing radiation could upregulate the glutamine metabolic flux and enhance ROS accumulation in mitochondria. Ionizing radiation was also found to decrease SLC7A11 expression, which resulted in reduced GSH generation. In this way, inhibition of mitophagy can increase ionizing radiation-induced cell death.

## Data Availability

All data generated and analyzed during this study are included in this article. Each experiment was performed at least three times independently.

## Supplementary Materials

Supplementary 1 Supplementary Table 1: list of antibodies and chemicals.

Supplementary 2 Supplementary Table 2: list of primers.

Supplementary 3 R language analysis code.

Supplementary 4 SLC7A11-BRCA-patient database.
