# Supplementary material for: Ionizing Radiation Upregulates Glutamine Metabolism and Induces Cell Death via Accumulation of Reactive Oxygen Species
Source: Oxid Med Cell Longev. 2021 Dec 30;2021:5826932. doi: 10.1155/2021/5826932 (PMC8749225; doi:10.1155/2021/5826932)
Supplement: Supplementary 1 — Supplementary Table 1: list of antibodies and chemicals. [file 5826932.f1.docx]

**Supplementary table 1. List of antibodies and Chemicals**

| **REAGENT SOURCE IDENTIFIER** | | |
| --- | --- | --- |
| **Antibodies**  **SLC7A11**  **SLC3A2**  **SLC1A5**  **SLC7A5**  **TOM20**  **Parkin**  **LC3B**  **p62**  **Beclin1**  **lamp2**  **c-Myc**  **γ-H2AX**  **Ki67**  **MDA**  **Ki67**  **Chemicals**  **Chloroquine (CQ)**  **Mdivi-1**  **EGCG** | **Proteintech**  **Abcam**  **Proteintech**  **Santa Cruz BioTech**  **Proteintech**  **Proteintech**  **Abcam**  **Abcam**  **Abcam**  **Abcam**  **Abcam**  **Abcam**  **Abcam**  **Servicebio**  **Servicebio**  **MedChemExpress**  **MedChemExpress**  **Beyotime** | **26864-1-AP**  **ab244356**  **20350-1-AP**  **sc-374232**  **11802-1-AP**  **66674-1-Ig**  **ab192890**  **ab109012**  **ab207612**  **ab199946**  **ab32072**  **ab26350**  **ab16667**  **gb11144**  **gb111141**  **HY-17589A**  **HY-15886**  **ST1011** |
