# Supplementary material for: Ionizing Radiation Upregulates Glutamine Metabolism and Induces Cell Death via Accumulation of Reactive Oxygen Species
Source: Oxid Med Cell Longev. 2021 Dec 30;2021:5826932. doi: 10.1155/2021/5826932 (PMC8749225; doi:10.1155/2021/5826932)
Supplement: Supplementary 2 — Supplementary Table 2: list of primers. [file 5826932.f2.docx]

**Supplementary table 2. List of primers**

| **Primers** | **Sequence** |
| --- | --- |
| **β-actin** | **F: TGGTATCGTGGAAGGACTC**  **R: AGTAGAGGCAGGGATGATG** |
| **ASCT2** | **F: CAGTCCTTGGACTTCGTAAAGA**  **R: CCAGGATCAAGGAGATATGGTC** |
| **GLUD1** | **F: CGGCAGAGTTCCAAGACAGGATA**  **R: AGGCAGCTGTTCTCAGGTCCA** |
| **GLS** | **F: GAAGGCACAGACATGGTTGGTA**  **R: ATTGGGCAGAAACCACCATTAG** |
