# Supplementary material for: Ionizing Radiation Upregulates Glutamine Metabolism and Induces Cell Death via Accumulation of Reactive Oxygen Species
Source: Oxid Med Cell Longev. 2021 Dec 30;2021:5826932. doi: 10.1155/2021/5826932 (PMC8749225; doi:10.1155/2021/5826932)
Supplement: Supplementary 3 — R language analysis code. [file 5826932.f3.docx]

library(Rcpp)

library("readr")

mydata<- read.csv('D:\\Desktop\\SLC7A11-BRCA_5071_50_50.csv',header = T)

library("survival")

library("survminer")

table(mydata$Status)

mydata$Status=ifelse(mydata$Status=='Dead',1,0)

sfit <- survfit(Surv(Days,Status)~Group, data=mydata)

summary(sfit)

#1

library(ggplot2)

ggsurvplot(sfit,

pval = TRUE, conf.int = TRUE,

risk.table = TRUE, # Add risk table

risk.table.col = "Group", # Change risk table color by groups

linetype = "Group", # Change line type by groups

surv.median.line = "hv", # Specify median survival

ggtheme = theme_bw(), # Change ggplot2 theme

palette = c("#E7B800", "#2E9FDF")

)
