# Supplementary material for: Ionizing Radiation Upregulates Glutamine Metabolism and Induces Cell Death via Accumulation of Reactive Oxygen Species
Source: Oxid Med Cell Longev. 2021 Dec 30;2021:5826932. doi: 10.1155/2021/5826932 (PMC8749225; doi:10.1155/2021/5826932)
Supplement: Supplementary 4 — SLC7A11-BRCA-patient database. [file 5826932.f4.docx]

Patient Days Status Expression Group

TCGA-OL-A66O 528 Alive 2.9 Low

TCGA-V7-A7HQ 2033 Alive 4.93 Low

TCGA-EW-A6SA 510 Alive 4.97 Low

TCGA-A2-A3XV 996 Alive 6.02 Low

TCGA-E9-A54Y 725 Alive 6.52 Low

TCGA-B6-A0RO 4929 Alive 6.58 Low

TCGA-C8-A12U 385 Alive 7.35 Low

TCGA-A7-A425 447 Alive 8.25 Low

TCGA-A7-A4SF 545 Alive 8.92 Low

TCGA-AN-A0FT 214 Alive 9.55 Low

TCGA-A2-A0CK 4159 Alive 9.7 Low

TCGA-A7-A426 364 Alive 9.71 Low

TCGA-AC-A3QQ 734 Alive 9.98 Low

TCGA-A7-A56D 448 Alive 10.01 Low

TCGA-OL-A66L 1301 Alive 10.45 Low

TCGA-E9-A249 217 Alive 10.55 Low

TCGA-AC-A5XS 588 Alive 10.68 Low

TCGA-GM-A4E0 2191 Alive 11.01 Low

TCGA-AN-A0FF 172 Alive 11.41 Low

TCGA-AN-A0XT 10 Alive 11.65 Low

TCGA-BH-A0E7 1363 Alive 11.8 Low

TCGA-AR-A5QN 1013 Alive 12.12 Low

TCGA-E2-A105 1308 Alive 12.82 Low

TCGA-GM-A3XN 2019 Alive 13.13 Low

TCGA-E2-A10C 1220 Alive 13.32 Low

TCGA-E9-A5UO 785 Alive 13.76 Low

TCGA-LQ-A4E4 849 Alive 14.46 Low

TCGA-E2-A1IG 2140 Alive 14.62 Low

TCGA-E9-A1RE 1419 Alive 14.88 Low

TCGA-E9-A5FK 812 Alive 15.24 Low

TCGA-A8-A06Q 31 Alive 15.43 Low

TCGA-AN-A0FZ 10 Alive 16.1 Low

TCGA-E2-A15H 393 Alive 16.5 Low

TCGA-B6-A2IU 5176 Alive 16.53 Low

TCGA-BH-A0BP 2296 Dead 16.55 Low

TCGA-AC-A2QI 588 Alive 16.84 Low

TCGA-AR-A5QP 1185 Alive 16.84 Low

TCGA-E2-A15O 1545 Alive 16.89 Low

TCGA-EW-A1P6 562 Alive 17.46 Low

TCGA-E2-A155 640 Alive 17.78 Low

TCGA-A2-A04Y 1099 Alive 19.06 Low

TCGA-BH-A0BM 1876 Alive 19.11 Low

TCGA-A2-A0D4 767 Alive 19.44 Low

TCGA-BH-A0AY 777 Alive 20.43 Low

TCGA-E2-A576 1043 Alive 20.62 Low

TCGA-BH-A0BT 2365 Alive 20.71 Low

TCGA-BH-A1EN 2127 Dead 20.96 Low

TCGA-C8-A138 380 Alive 21.57 Low

TCGA-AC-A23G 2248 Alive 21.8 Low

TCGA-A2-A25C 523 Alive 21.84 Low

TCGA-AC-A2FM 792 Dead 22.05 Low

TCGA-BH-A0HA 1611 Alive 22.27 Low

TCGA-B6-A40B 3152 Alive 22.4 Low

TCGA-AR-A2LO 1198 Alive 22.6 Low

TCGA-JL-A3YX 352 Alive 22.61 Low

TCGA-3C-AALI 4005 Alive 22.84 Low

TCGA-BH-A0H6 747 Alive 23.11 Low

TCGA-A2-A0CP 2813 Alive 23.46 Low

TCGA-A8-A084 458 Alive 23.59 Low

TCGA-LL-A50Y 762 Alive 23.82 Low

TCGA-AC-A3TN 456 Alive 24 Low

TCGA-LD-A66U 646 Alive 24.34 Low

TCGA-BH-A208 1759 Dead 24.84 Low

TCGA-AC-A3HN 496 Alive 25.02 Low

TCGA-E2-A1LA 748 Alive 25.37 Low

TCGA-BH-A42V 635 Alive 25.61 Low

TCGA-AQ-A04L 3957 Alive 25.63 Low

TCGA-AC-A3BB 987 Alive 25.7 Low

TCGA-D8-A1JU 447 Alive 25.93 Low

TCGA-E2-A106 2541 Alive 26.08 Low

TCGA-S3-A6ZH 641 Alive 26.14 Low

TCGA-AC-A3TM 762 Alive 26.79 Low

TCGA-LL-A9Q3 532 Alive 26.79 Low

TCGA-BH-AB28 287 Alive 27.1 Low

TCGA-AN-A0FW 11 Alive 27.14 Low

TCGA-A7-A0DB 1007 Alive 27.21 Low

TCGA-A1-A0SI 635 Alive 27.37 Low

TCGA-E9-A1R4 186 Alive 27.42 Low

TCGA-D8-A140 403 Alive 27.47 Low

TCGA-A2-A0YE 554 Alive 27.57 Low

TCGA-AR-A24R 3430 Alive 27.61 Low

TCGA-LD-A7W5 216 Alive 28.21 Low

TCGA-BH-A1F5 2712 Dead 28.37 Low

TCGA-BH-A0BV 1519 Alive 28.4 Low

TCGA-B6-A0RM 2373 Dead 28.89 Low

TCGA-BH-A0C7 2767 Alive 28.92 Low

TCGA-A2-A3XZ 1532 Alive 29.29 Low

TCGA-A2-A0CW 3283 Alive 29.31 Low

TCGA-E9-A1RG 647 Alive 29.57 Low

TCGA-AR-A0TY 1699 Dead 29.67 Low

TCGA-A8-A075 518 Alive 29.76 Low

TCGA-BH-A0HU 392 Alive 30.24 Low

TCGA-BH-A28O 1120 Alive 30.52 Low

TCGA-HN-A2OB 1900 Dead 30.58 Low

TCGA-B6-A0I8 749 Dead 30.65 Low

TCGA-E2-A154 591 Alive 30.82 Low

TCGA-A8-A08B 1156 Alive 30.92 Low

TCGA-AR-A2LJ 2632 Alive 30.97 Low

TCGA-C8-A26Z 470 Alive 31.45 Low

TCGA-B6-A0WZ 6292 Alive 31.49 Low

TCGA-LL-A6FP 677 Alive 31.59 Low

TCGA-A8-A076 1642 Alive 31.7 Low

TCGA-GM-A2DC 2535 Alive 31.73 Low

TCGA-A2-A3XX 1439 Dead 31.87 Low

TCGA-Z7-A8R5 3287 Alive 32.26 Low

TCGA-E9-A22D 1248 Alive 32.74 Low

TCGA-D8-A1JB 1688 Alive 32.78 Low

TCGA-A2-A04N 4354 Alive 32.88 Low

TCGA-AO-A0J9 1613 Alive 32.97 Low

TCGA-BH-A0DV 2064 Alive 33.03 Low

TCGA-WT-AB41 1611 Alive 33.12 Low

TCGA-D8-A1JH 426 Alive 33.14 Low

TCGA-BH-A0B7 2559 Alive 33.24 Low

TCGA-BH-A0BJ 660 Alive 33.35 Low

TCGA-A2-A0EO 2442 Alive 33.36 Low

TCGA-C8-A273 513 Alive 33.44 Low

TCGA-LL-A6FQ 80 Alive 33.91 Low

TCGA-A8-A08L 304 Dead 33.95 Low

TCGA-C8-A274 508 Alive 34.02 Low

TCGA-AN-A041 7 Alive 34.2 Low

TCGA-AC-A3EH 197 Dead 34.21 Low

TCGA-A8-A0A2 579 Alive 34.22 Low

TCGA-A2-A0T2 255 Dead 34.33 Low

TCGA-B6-A0I9 362 Dead 34.42 Low

TCGA-A2-A0D3 1873 Alive 34.5 Low

TCGA-BH-A5J0 715 Alive 34.75 Low

TCGA-BH-A18M 2207 Dead 34.89 Low

TCGA-E2-A1LE 879 Dead 35.14 Low

TCGA-A2-A3XU 912 Dead 35.31 Low

TCGA-D8-A27N 519 Alive 35.42 Low

TCGA-E2-A1LS 1604 Alive 35.54 Low

TCGA-LL-A5YM 466 Alive 35.66 Low

TCGA-B6-A40C 2164 Alive 35.67 Low

TCGA-BH-A1EV 365 Dead 35.7 Low

TCGA-E2-A107 1047 Alive 35.7 Low

TCGA-E2-A1BC 501 Alive 35.8 Low

TCGA-PE-A5DE 2645 Alive 35.98 Low

TCGA-A8-A07E 608 Alive 36.1 Low

TCGA-LL-A6FR 489 Alive 36.12 Low

TCGA-E9-A3X8 926 Alive 36.17 Low

TCGA-GM-A2DK 2645 Alive 36.21 Low

TCGA-D8-A1Y1 302 Dead 36.56 Low

TCGA-A2-A0EY 1925 Alive 36.59 Low

TCGA-AC-A6IV 568 Alive 36.84 Low

TCGA-AO-A03P 2911 Dead 37.06 Low

TCGA-3C-AAAU 4047 Alive 37.57 Low

TCGA-D8-A27K 1461 Alive 37.77 Low

TCGA-A2-A0CR 3283 Alive 38.35 Low

TCGA-A2-A0SV 825 Dead 39.1 Low

TCGA-AR-A1AN 2920 Alive 39.13 Low

TCGA-A2-A25D 552 Alive 39.22 Low

TCGA-C8-A8HP 396 Alive 39.5 Low

TCGA-C8-A12M 358 Alive 39.7 Low

TCGA-A2-A3KC 1102 Alive 39.77 Low

TCGA-BH-A1ET 2520 Dead 40.03 Low

TCGA-E2-A14P 1246 Alive 40.05 Low

TCGA-EW-A1OV 789 Alive 40.08 Low

TCGA-AQ-A7U7 584 Dead 40.6 Low

TCGA-BH-A0BQ 2255 Alive 40.6 Low

TCGA-BH-A8G0 662 Alive 40.77 Low

TCGA-PE-A5DC 1430 Dead 40.78 Low

TCGA-AC-A2BK 2222 Alive 40.93 Low

TCGA-AO-A12A 3112 Alive 40.94 Low

TCGA-C8-A1HI 343 Alive 41.15 Low

TCGA-E2-A15C 694 Alive 41.2 Low

TCGA-AC-A3QP 675 Alive 41.27 Low

TCGA-BH-A42U 3364 Alive 41.44 Low

TCGA-A2-A0T7 631 Alive 41.6 Low

TCGA-PL-A8LY 8 Alive 41.6 Low

TCGA-BH-A0EA 991 Dead 41.63 Low

TCGA-B6-A0IH 3418 Dead 41.73 Low

TCGA-E2-A1IJ 865 Alive 41.75 Low

TCGA-LL-A5YN 447 Alive 41.8 Low

TCGA-A1-A0SQ 554 Alive 41.96 Low

TCGA-OL-A5D7 1780 Alive 42.05 Low

TCGA-AO-A03N 2031 Alive 42.06 Low

TCGA-BH-A0DT 2403 Alive 42.18 Low

TCGA-AN-A0AS 10 Alive 42.35 Low

TCGA-A1-A0SJ 416 Alive 42.43 Low

TCGA-OL-A5RW 1106 Alive 42.69 Low

TCGA-AO-A12D 2515 Alive 42.77 Low

TCGA-S3-AA0Z 629 Alive 42.82 Low

TCGA-AR-A0TZ 3262 Dead 43.07 Low

TCGA-WT-AB44 883 Alive 43.17 Low

TCGA-A7-A13G 718 Alive 43.32 Low

TCGA-B6-A0WT 5739 Alive 43.51 Low

TCGA-C8-A8HR 408 Alive 43.65 Low

TCGA-E9-A1R0 860 Alive 43.7 Low

TCGA-AC-A4ZE 890 Alive 43.72 Low

TCGA-BH-A1EY 538 Dead 43.88 Low

TCGA-OL-A66K 1275 Dead 43.93 Low

TCGA-B6-A0I5 8556 Alive 44.24 Low

TCGA-C8-A3M7 1034 Dead 44.29 Low

TCGA-A2-A0CV 3011 Alive 44.41 Low

TCGA-PL-A8LX 5 Alive 44.51 Low

TCGA-AO-A0JM 2184 Alive 44.66 Low

TCGA-BH-A18F 1001 Alive 44.72 Low

TCGA-D8-A1JG 1612 Alive 44.76 Low

TCGA-BH-A1FG 3736 Dead 45.16 Low

TCGA-E2-A1IL 118 Alive 45.37 Low

TCGA-BH-A6R8 293 Alive 45.54 Low

TCGA-AC-A8OP 614 Alive 45.55 Low

TCGA-D8-A3Z5 1015 Alive 45.55 Low

TCGA-AO-A0J2 997 Alive 45.59 Low

TCGA-B6-A0RQ 4267 Dead 45.82 Low

TCGA-BH-A0DH 1156 Alive 45.92 Low

TCGA-AN-A03X 10 Alive 45.93 Low

TCGA-A2-A25A 3276 Alive 45.97 Low

TCGA-OK-A5Q2 64 Alive 46.03 Low

TCGA-OL-A5D6 1104 Dead 46.13 Low

TCGA-A2-A04X 1686 Alive 46.5 Low

TCGA-AR-A2LN 1161 Alive 46.73 Low

TCGA-E2-A1BD 1133 Alive 46.8 Low

TCGA-AN-A0FD 196 Alive 47.04 Low

TCGA-C8-A26Y 394 Alive 47.12 Low

TCGA-A2-A0CZ 1616 Alive 47.14 Low

TCGA-A1-A0SN 1196 Alive 47.15 Low

TCGA-XX-A89A 488 Alive 47.24 Low

TCGA-EW-A6SD 1010 Alive 47.34 Low

TCGA-A8-A08X 1308 Alive 47.44 Low

TCGA-OL-A5RZ 679 Alive 47.57 Low

TCGA-A2-A0T5 531 Alive 47.78 Low

TCGA-AN-A0XS 10 Alive 48.06 Low

TCGA-EW-A1OX 911 Alive 48.11 Low

TCGA-BH-A18G 149 Alive 48.13 Low

TCGA-OL-A66J 1996 Alive 48.22 Low

TCGA-E9-A3HO 1158 Alive 48.3 Low

TCGA-BH-A0B5 2136 Alive 48.7 Low

TCGA-BH-A1FL 1673 Dead 48.75 Low

TCGA-B6-A0IG 4456 Dead 48.86 Low

TCGA-E9-A2JT 288 Alive 48.87 Low

TCGA-EW-A6SC 952 Alive 49.12 Low

TCGA-A2-A0CO 3492 Dead 49.3 Low

TCGA-E2-A14O 1359 Alive 49.3 Low

TCGA-OL-A66N 792 Alive 49.39 Low

TCGA-3C-AALK 1448 Alive 49.65 Low

TCGA-E2-A15R 1732 Alive 49.77 Low

TCGA-AC-A2FK 2650 Alive 49.8 Low

TCGA-BH-A0AZ 1919 Alive 49.83 Low

TCGA-D8-A142 425 Alive 49.93 Low

TCGA-BH-A0E0 134 Alive 49.97 Low

TCGA-BH-A0W7 1363 Alive 50.13 Low

TCGA-A2-A0YK 588 Alive 50.21 Low

TCGA-E2-A1LG 1523 Alive 50.37 Low

TCGA-AC-A2FE 2636 Dead 50.44 Low

TCGA-A2-A0D1 1051 Alive 50.85 Low

TCGA-BH-A0BO 2197 Alive 51.01 Low

TCGA-A2-A0EN 4088 Alive 51.15 Low

TCGA-OL-A5D8 973 Alive 51.28 Low

TCGA-BH-A0BZ 2255 Alive 51.32 Low

TCGA-BH-A0E6 293 Alive 51.4 Low

TCGA-B6-A409 573 Dead 209.86 High

TCGA-C8-A12Q 385 Dead 210.16 High

TCGA-C8-A8HQ 380 Alive 210.34 High

TCGA-BH-A1ES 3462 Dead 212.3 High

TCGA-A7-A6VY 266 Alive 213.08 High

TCGA-BH-A0HK 178 Alive 213.38 High

TCGA-AR-A1AI 3296 Alive 213.41 High

TCGA-A2-A25E 3204 Alive 213.84 High

TCGA-LL-A5YP 450 Alive 216.55 High

TCGA-UU-A93S 116 Dead 217.08 High

TCGA-A1-A0SO 852 Alive 217.9 High

TCGA-A7-A3J0 313 Alive 218.44 High

TCGA-E2-A1LI 3121 Alive 219.05 High

TCGA-AR-A1AO 2618 Alive 219.47 High

TCGA-AR-A1AX 2629 Alive 220.04 High

TCGA-B6-A0IJ 7106 Alive 220.87 High

TCGA-AO-A0JD 2190 Alive 222.37 High

TCGA-AQ-A04J 819 Alive 229.12 High

TCGA-UL-AAZ6 518 Alive 229.95 High

TCGA-C8-A1HN 394 Alive 230.06 High

TCGA-AR-A1AK 3159 Alive 231.3 High

TCGA-C8-A12O 385 Alive 231.46 High

TCGA-A8-A07B 1308 Alive 231.61 High

TCGA-C8-A130 370 Alive 234.91 High

TCGA-D8-A27G 409 Alive 235.35 High

TCGA-GM-A2DB 2406 Alive 236.39 High

TCGA-A8-A0A6 640 Alive 238.33 High

TCGA-GM-A2DL 3519 Alive 238.43 High

TCGA-EW-A1PD 424 Alive 239.01 High

TCGA-D8-A1JN 620 Alive 239.39 High

TCGA-B6-A0X4 860 Dead 240.26 High

TCGA-A8-A0A1 365 Alive 240.96 High

TCGA-D8-A1J9 532 Alive 242.02 High

TCGA-EW-A1J3 504 Alive 242.15 High

TCGA-A8-A07J 365 Alive 242.9 High

TCGA-BH-A18S 2009 Dead 245.27 High

TCGA-E2-A15J 1640 Alive 245.94 High

TCGA-A2-A3XY 1093 Dead 247.48 High

TCGA-E2-A1L8 2240 Alive 247.71 High

TCGA-EW-A6S9 463 Alive 250.86 High

TCGA-A8-A08T 3409 Dead 250.92 High

TCGA-E9-A2JS 904 Dead 251.29 High

TCGA-A2-A4S1 820 Alive 252.7 High

TCGA-E2-A15L 626 Alive 255.52 High

TCGA-E9-A54X 727 Alive 257.7 High

TCGA-BH-A18T 224 Dead 258.88 High

TCGA-BH-A0BL 2278 Alive 261.7 High

TCGA-E9-A1R5 92 Alive 262.4 High

TCGA-AN-A0AT 10 Alive 263.19 High

TCGA-AN-A0AL 227 Alive 264.9 High

TCGA-AO-A0JE 2335 Alive 266.13 High

TCGA-A8-A08P 943 Alive 266.18 High

TCGA-AR-A0U4 3261 Alive 266.23 High

TCGA-A7-A2KD 679 Alive 266.36 High

TCGA-E2-A15A 710 Alive 267.13 High

TCGA-AQ-A0Y5 172 Dead 267.23 High

TCGA-E9-A22G 1239 Alive 272.41 High

TCGA-BH-A0B3 1203 Alive 274.11 High

TCGA-A8-A099 304 Alive 274.15 High

TCGA-E2-A1IK 1800 Alive 276.11 High

TCGA-B6-A400 215 Alive 276.39 High

TCGA-BH-A203 1174 Dead 277.11 High

TCGA-AR-A255 2161 Alive 277.77 High

TCGA-BH-A1F0 785 Dead 278.09 High

TCGA-E2-A10E 865 Alive 279.05 High

TCGA-D8-A1XT 506 Alive 279.12 High

TCGA-A7-A0DA 1085 Alive 281.12 High

TCGA-E2-A14S 1009 Alive 282.96 High

TCGA-A2-A1G1 584 Alive 283.69 High

TCGA-EW-A1PB 608 Alive 284.32 High

TCGA-B6-A402 2134 Alive 287.28 High

TCGA-C8-A12V 385 Alive 287.92 High

TCGA-E2-A1L7 1836 Alive 288.73 High

TCGA-BH-A0AV 1820 Alive 290.46 High

TCGA-A2-A3Y0 1546 Alive 291.25 High

TCGA-A7-A0DC 906 Alive 293.99 High

TCGA-E2-A15F 658 Alive 294.5 High

TCGA-AR-A0U3 4080 Alive 294.92 High

TCGA-EW-A423 533 Alive 295.13 High

TCGA-A8-A07G 577 Alive 296.36 High

TCGA-BH-A0B1 1148 Alive 298.06 High

TCGA-A8-A07W 304 Alive 298.09 High

TCGA-B6-A0WY 3461 Dead 299.62 High

TCGA-B6-A0RS 3063 Dead 302.4 High

TCGA-A8-A07U 760 Alive 303.54 High

TCGA-BH-A0EE 943 Alive 307.62 High

TCGA-D8-A145 410 Alive 308.31 High

TCGA-E9-A24A 747 Alive 308.34 High

TCGA-A2-A0T3 1516 Alive 309.71 High

TCGA-AR-A1AR 524 Dead 310.18 High

TCGA-A7-A4SE 644 Alive 310.88 High

TCGA-BH-A201 856 Alive 311.16 High

TCGA-AN-A0AR 10 Alive 312.69 High

TCGA-BH-A202 795 Alive 313.77 High

TCGA-S3-AA10 586 Alive 313.91 High

TCGA-BH-A18P 921 Dead 314.57 High

TCGA-A7-A26H 724 Alive 314.87 High

TCGA-E2-A14Y 2109 Alive 319.22 High

TCGA-BH-A0HP 414 Alive 320.56 High

TCGA-BH-A0DD 2486 Alive 320.73 High

TCGA-A2-A259 1596 Alive 321.31 High

TCGA-EW-A1J6 875 Alive 323.91 High

TCGA-GM-A2DM 3226 Alive 327.88 High

TCGA-AO-A03V 1351 Alive 328.24 High

TCGA-GM-A2DH 2193 Alive 329.07 High

TCGA-AN-A046 10 Alive 330.56 High

TCGA-B6-A0IB 3941 Dead 331.61 High

TCGA-EW-A1PF 439 Alive 331.77 High

TCGA-EW-A1OW 694 Alive 333.53 High

TCGA-E2-A10A 1229 Alive 338.16 High

TCGA-AO-A0J4 1587 Alive 338.28 High

TCGA-AC-A2BM 3022 Alive 339.83 High

TCGA-BH-A1EW 1694 Dead 342.2 High

TCGA-D8-A1JE 575 Alive 343 High

TCGA-B6-A0IK 571 Dead 345.09 High

TCGA-E2-A1LK 266 Dead 345.1 High

TCGA-A2-A0ET 1066 Alive 345.88 High

TCGA-AR-A24V 3203 Alive 345.97 High

TCGA-E9-A1N8 1039 Alive 347.11 High

TCGA-C8-A27B 439 Alive 349.78 High

TCGA-BH-A0BW 2371 Alive 352.62 High

TCGA-A8-A07C 1034 Alive 352.93 High

TCGA-E9-A1RD 34 Alive 353.76 High

TCGA-A2-A0YJ 566 Alive 354.35 High

TCGA-A2-A3XS 1032 Dead 355.55 High

TCGA-A8-A08S 1004 Alive 357.1 High

TCGA-A2-A0CQ 2695 Alive 359.77 High

TCGA-AR-A24Q 3172 Alive 360.7 High

TCGA-D8-A1JL 611 Alive 360.81 High

TCGA-BH-A0BD 554 Alive 361.67 High

TCGA-A2-A0EQ 2426 Alive 361.87 High

TCGA-D8-A1X6 541 Alive 363.55 High

TCGA-A8-A06R 547 Alive 365.41 High

TCGA-C8-A12L 363 Alive 367.23 High

TCGA-C8-A1HJ 5 Alive 370.1 High

TCGA-EW-A1IY 258 Alive 370.22 High

TCGA-D8-A27I 439 Alive 377.15 High

TCGA-A7-A13E 614 Dead 377.5 High

TCGA-BH-A0HF 727 Alive 378.83 High

TCGA-D8-A1XA 839 Alive 380.51 High

TCGA-AC-A23C 585 Alive 381.11 High

TCGA-D8-A1JD 552 Alive 381.93 High

TCGA-LL-A442 889 Alive 382.07 High

TCGA-A2-A0YG 666 Alive 386.87 High

TCGA-BH-A204 2534 Dead 402.45 High

TCGA-EW-A1P4 907 Alive 403.19 High

TCGA-AR-A256 2854 Dead 405.95 High

TCGA-D8-A1JA 502 Alive 406.82 High

TCGA-BH-A0HO 76 Alive 407.52 High

TCGA-A8-A06X 943 Dead 408.34 High

TCGA-B6-A0I6 991 Dead 409.89 High

TCGA-A2-A1G4 595 Alive 410 High

TCGA-BH-A0HW 1561 Alive 413.99 High

TCGA-BH-A0E2 435 Alive 433.67 High

TCGA-BH-A0EI 1926 Alive 437.62 High

TCGA-A1-A0SP 584 Alive 441.81 High

TCGA-AC-A3OD 451 Alive 448.28 High

TCGA-B6-A0RL 2469 Dead 448.62 High

TCGA-C8-A134 383 Alive 451.72 High

TCGA-AN-A0XN 10 Alive 451.81 High

TCGA-AO-A128 3248 Alive 453.46 High

TCGA-E9-A3Q9 1001 Alive 460.46 High

TCGA-BH-A1FJ 1927 Dead 460.56 High

TCGA-AO-A1KS 350 Alive 462.7 High

TCGA-A2-A0CM 754 Dead 462.91 High

TCGA-A2-A0YC 990 Alive 466.69 High

TCGA-A7-A3IY 345 Alive 467.01 High

TCGA-D8-A73W 385 Dead 476.59 High

TCGA-BH-A0AU 1914 Alive 482.4 High

TCGA-AN-A0AM 5 Alive 487.8 High

TCGA-A2-A0CU 158 Dead 497.9 High

TCGA-E2-A14N 1434 Alive 498.54 High

TCGA-E9-A3QA 918 Alive 502.81 High

TCGA-A7-A4SC 446 Alive 504.25 High

TCGA-A7-A3IZ 322 Alive 505.58 High

TCGA-A2-A0T4 624 Alive 507.56 High

TCGA-A7-A13F 765 Alive 512.45 High

TCGA-A2-A1FW 528 Alive 514.5 High

TCGA-BH-A1F8 763 Dead 516.2 High

TCGA-E9-A1R6 339 Alive 526.51 High

TCGA-D8-A1XG 448 Alive 533.36 High

TCGA-GM-A3NW 3361 Alive 542.5 High

TCGA-D8-A1JF 366 Alive 544.96 High

TCGA-D8-A1JC 480 Alive 559.92 High

TCGA-AN-A0XU 10 Alive 564.78 High

TCGA-A8-A06U 883 Dead 569.04 High

TCGA-C8-A26V 616 Alive 569.26 High

TCGA-A2-A0YM 965 Alive 570.72 High

TCGA-AN-A03Y 10 Alive 604.14 High

TCGA-AR-A1AP 2856 Alive 605.03 High

TCGA-BH-A0DI 912 Alive 609.99 High

TCGA-A8-A07O 304 Alive 615.13 High

TCGA-A2-A1FV 714 Alive 619.03 High

TCGA-A2-A0D2 1027 Alive 621.7 High

TCGA-BH-A18H 652 Alive 633.54 High

TCGA-A8-A08I 365 Alive 637.05 High

TCGA-AN-A049 19 Alive 647.29 High

TCGA-C8-A137 379 Alive 654.28 High

TCGA-AR-A0TU 709 Alive 660.51 High

TCGA-BH-A1FM 1388 Dead 660.81 High

TCGA-A2-A25F 322 Alive 666.75 High

TCGA-AR-A24L 2866 Dead 669.62 High

TCGA-AR-A24S 2976 Alive 676.81 High

TCGA-A8-A06Z 31 Alive 688.49 High

TCGA-D8-A27R 307 Alive 688.66 High

TCGA-AR-A0U2 2551 Dead 689.66 High

TCGA-D8-A27M 410 Alive 689.96 High

TCGA-E2-A14W 974 Alive 690.94 High

TCGA-E9-A5FL 24 Alive 695.09 High

TCGA-D8-A1XK 441 Alive 705.18 High

TCGA-GI-A2C9 3342 Alive 712.64 High

TCGA-BH-A18V 1556 Dead 717.87 High

TCGA-E9-A226 1048 Dead 723 High

TCGA-BH-A0BC 974 Alive 737.92 High

TCGA-E9-A244 21 Alive 754.66 High

TCGA-GM-A3XL 2108 Alive 754.93 High

TCGA-E2-A152 2128 Alive 795.09 High

TCGA-D8-A1XC 377 Dead 795.38 High

TCGA-AQ-A1H2 475 Alive 807.58 High

TCGA-BH-A0DE 2372 Alive 816.05 High

TCGA-D8-A1XV 461 Alive 820.2 High

TCGA-AR-A2LR 1742 Alive 823.91 High

TCGA-D8-A27F 488 Alive 827.96 High

TCGA-BH-A1FD 1009 Dead 839.62 High

TCGA-AR-A24U 3128 Alive 850.39 High

TCGA-AN-A0AK 224 Alive 895.47 High

TCGA-A8-A07Z 1371 Alive 930.54 High

TCGA-AO-A0J6 1140 Alive 1005.94 High

TCGA-A2-A0CY 1673 Alive 1065.68 High

TCGA-AR-A24Z 3001 Alive 1077.94 High

TCGA-B6-A0X1 7455 Dead 1087.64 High

TCGA-AN-A0G0 16 Alive 1129.9 High

TCGA-AC-A23H 174 Dead 1134.86 High

TCGA-C8-A1HG 345 Alive 1175.22 High

TCGA-AR-A0U0 1988 Alive 1202.35 High

TCGA-A7-A26E 954 Alive 1210.23 High

TCGA-AC-A2QH 1005 Alive 1222.59 High

TCGA-BH-A1FN 2192 Dead 1415.42 High

TCGA-A8-A079 274 Alive 1490.55 High

TCGA-E2-A1LL 1309 Alive 1513.75 High

TCGA-EW-A1P5 703 Alive 1656.04 High

TCGA-BH-A0HX 829 Alive 1726.43 High

TCGA-AR-A0TV 2288 Alive 1854.39 High

TCGA-A8-A07R 273 Alive 2157.56 High

TCGA-D8-A1JI 577 Alive 2310.89 High

TCGA-D8-A1JJ 611 Alive 2431.65 High

TCGA-E2-A10B 1141 Alive 2770.12 High

TCGA-BH-A8FY 295 Dead 2924.96 High

TCGA-AR-A251 3030 Alive 2965.11 High

TCGA-B6-A0X5 2097 Dead 3470.41 High

TCGA-AO-A0JJ 1887 Alive 4513.13 High
